# Supplementary material for: Synthesis, Structural Elucidation and Pharmacological Applications of Cu(II) Heteroleptic Carboxylates
Source: Pharmaceuticals (Basel). 2023 May 3;16(5):693. doi: 10.3390/ph16050693 (PMC10224544; doi:10.3390/ph16050693)
Supplement: Supplementary file 1 [file pharmaceuticals-16-00693-s001.zip › pharmaceuticals-2332394-supplementary.pdf]

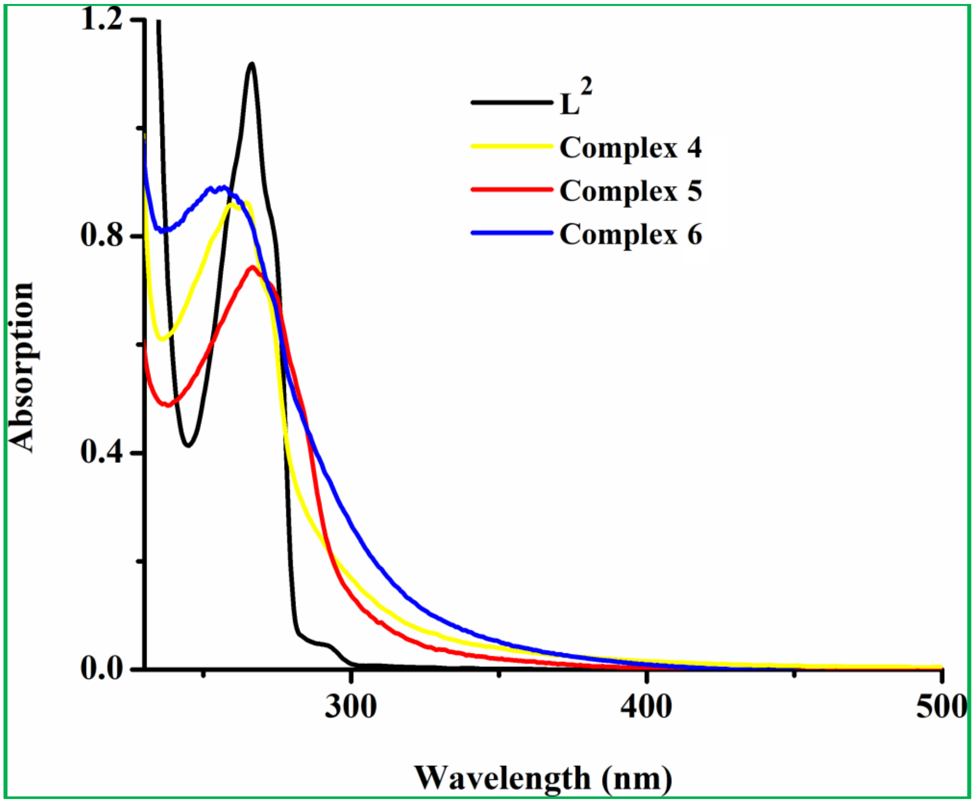

Figure S1. UV-Visible absorption spectrum of ligand **L²** and complexes **4-6** in ethanol.

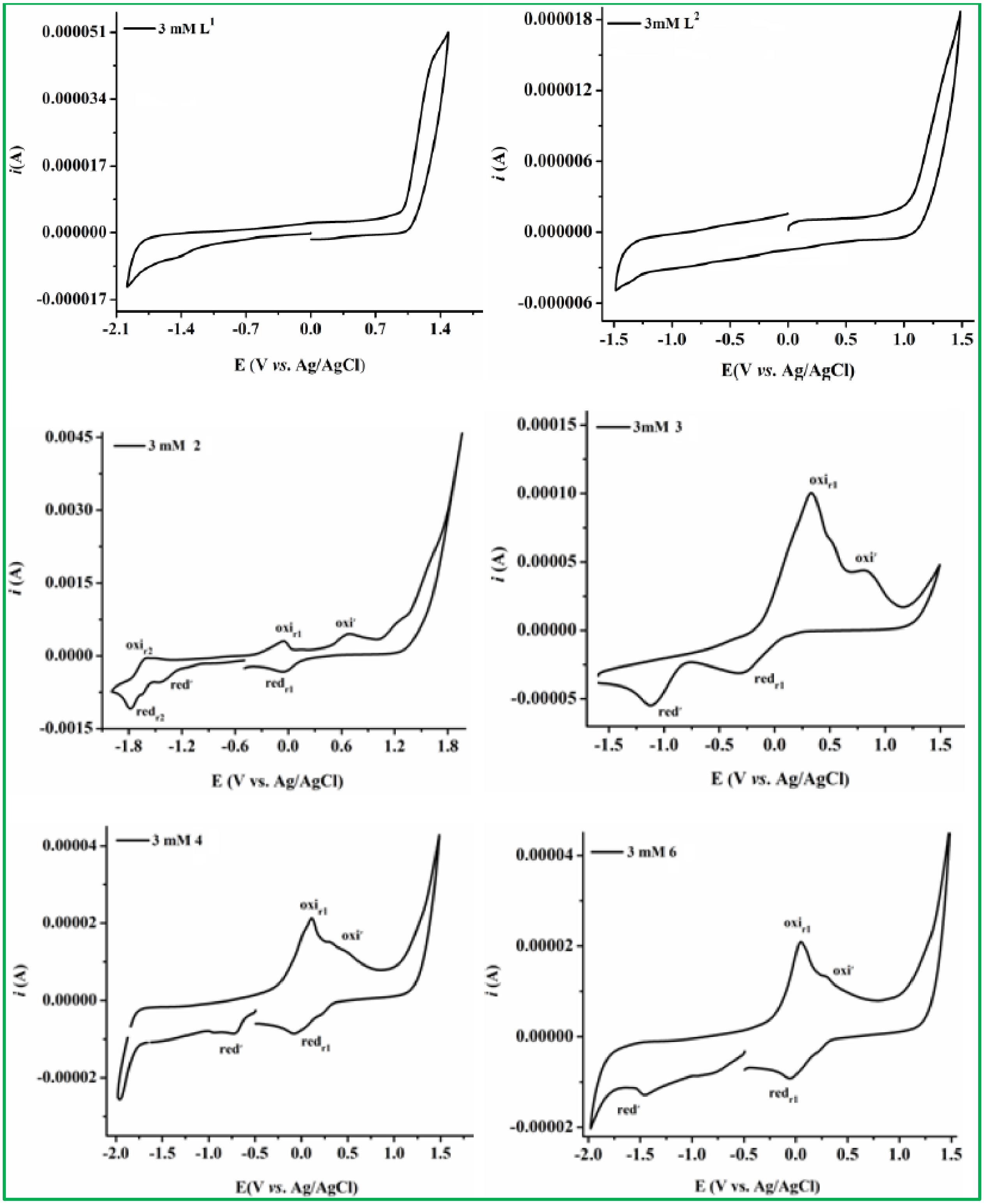

Figure S2. Cyclic voltammograms of Ligand **L**^{1}**, L**^{2} and complexes **2-4** and **6**

Table S1. Electrochemical parameter for complexes **1-6** before and after DNA addition

**Comp.**	**E (V) before DNA**			**E (V) after DNA**			**Change in potentials**		
	**Ea**	**Ec**	**$E_{1/2}$**	**Ea′**	**Ec′**	**$E_{1/2}$′**	**ΔEa**	**ΔEc**	**Δ$E_{1/2}$**
	0.478	n.o	-	0.535	n.o	-	0.056		
**1**	0.203	-0.268	-0.032	0.336	-0.158	0.088	0.132	0.109	0.012
	n.o	-1.016	-	n.o	-0.591		-	0.424	
	0.690	n.o		0.821	n.o		0.131		
**2**	-0.049	-0.055	-0.052	0.271	0.031	0.151	0.321	0.087	0.20
	n.o	-1.428		n.o	-1.145		-	0.283	
	-1.602	-1.780	-3.383	-1.413	-1.638	-1.525	0.189	0.142	1.85
	0.846	n.o		disappear	n.o		-	-	
**3**	0.326	-0.327	-5E-05	0.511	-0.267	0.121	0.184	0.059	1.21E-01
	n.o	-1.122		n.o	0.611			1.734	
	0.315	n.o		0.318	n.o		0.002		
**4**	0.112	-0.076	0.01785	0.134	-0.047	0.110	0.021	0.028	0.092
	n.O	-0.723		n.o	disappear				
	0.570	0.132		0.720	disappear		0.150		
**5**	0.134	0.067	0.1008	0.129	-0.050	0.039	-0.005	-0.117	-0.06
	-1.226	-1.422		disappear	-1.169			0.253	
	0.282	n.o		disappear	n.o				
**6**	0.071	-0.086	-0.0076	0.148	0.0036	0.076	0.076	0.090	0.08
	n.o	-0.982		n.o	disappear				
	n.o	-1.278		n.o	diappear				

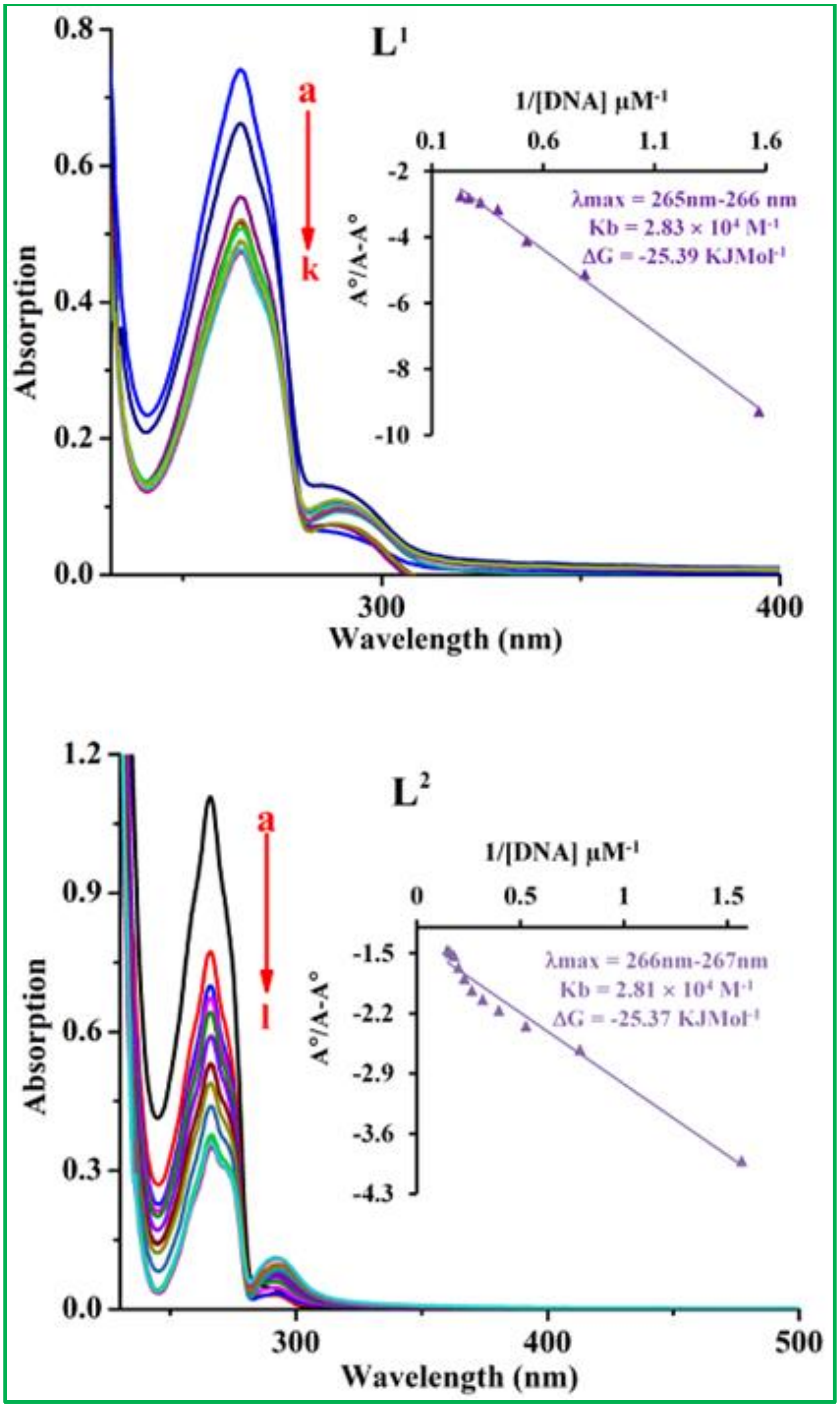

Figure S3. UV-visible absorption spectra of ligand acids **L**[1] and **L**[2] in the absence (a in each case) and in the presence of incremental addition, i.e., 0.63-6.631 μM (**L**[1]), and 0.63-4.41 μM (**L**[2]), respectively. Whereas the inside graph revels the values of Kb and ΔG calculated from graph plotted between the absorption and DNA concentration.

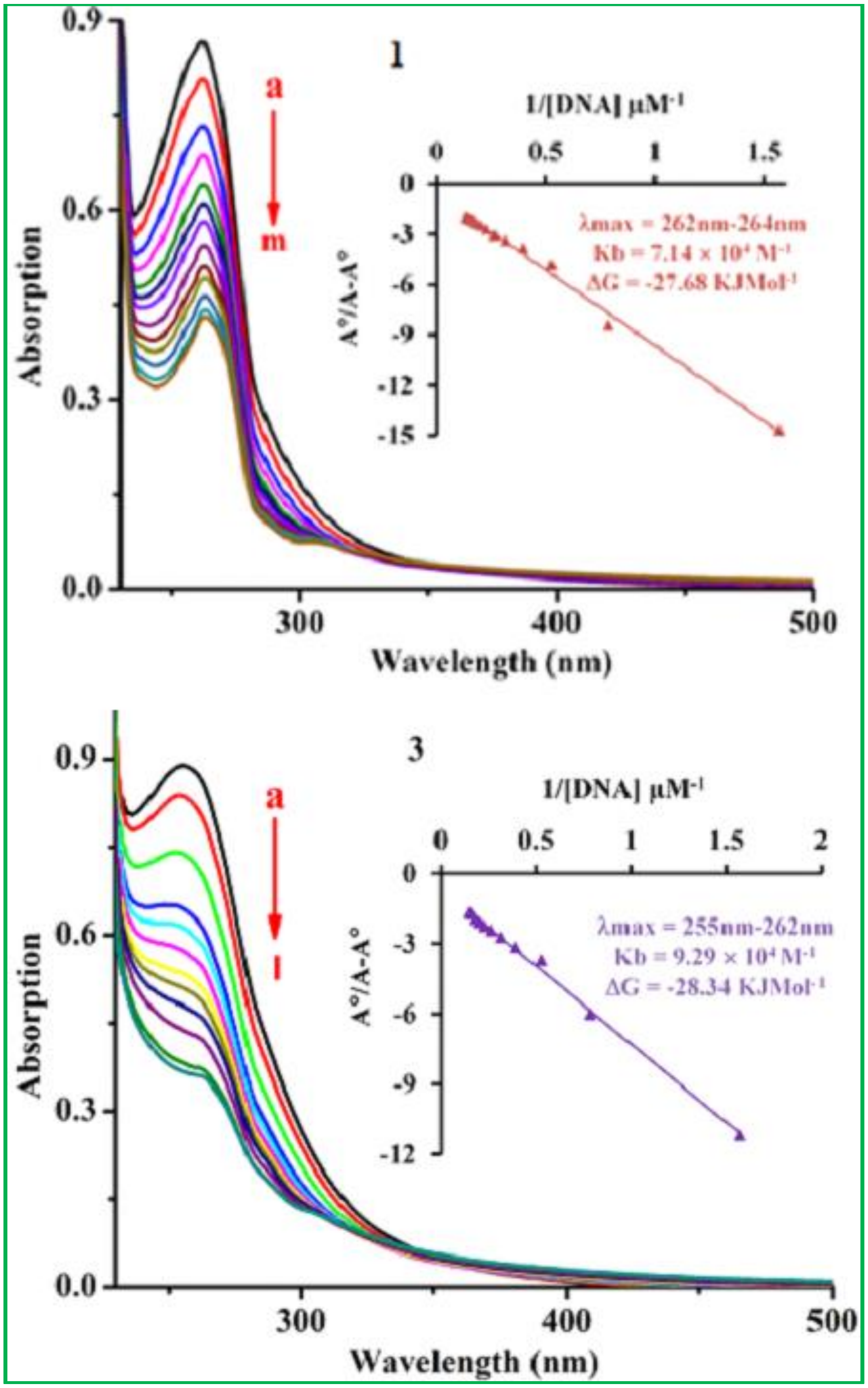

Figure S4. UV-visible absorption spectra of complexes **1** and **3** in the absence (a in each case) and in the presence of incremental addition, i.e., 0.63-8.19 μM (**1**), 0.63-5.67 μM (**3**, respectively. Whereas the inside graph revels the values of Kb and ΔG calculated from graph plotted between the absorption and DNA concentration.

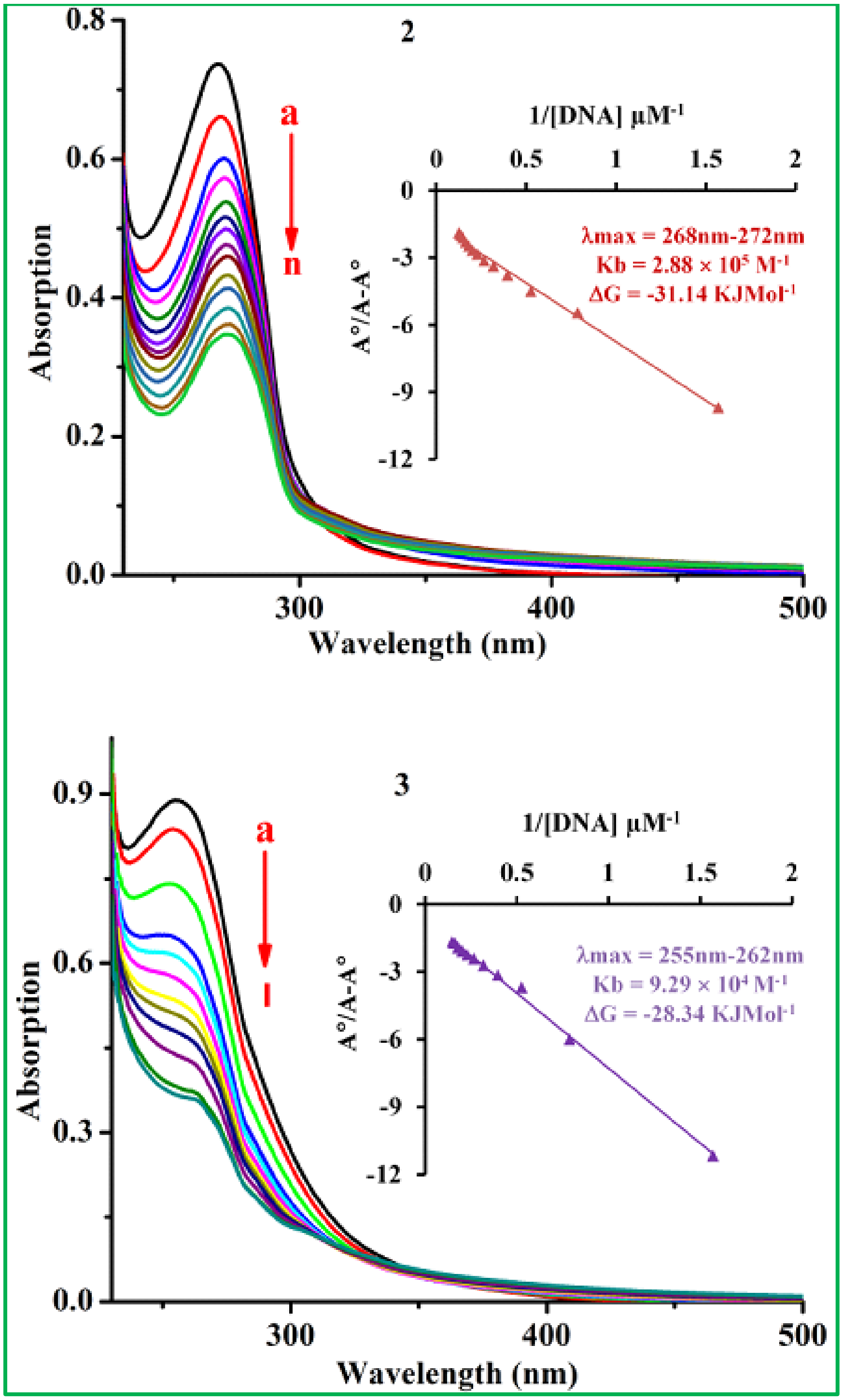

Figure S5. UV-visible absorption spectra of complexes **2** and **3** in the absence (a in each case) and in the presence of incremental addition, i.e., 0.63-8.19 μM (**2**), 0.63-5.04 μM (**3**, respectively. Whereas the inside graph revels the values of Kb and ΔG calculated from graph plotted between the absorption and DNA concentration.

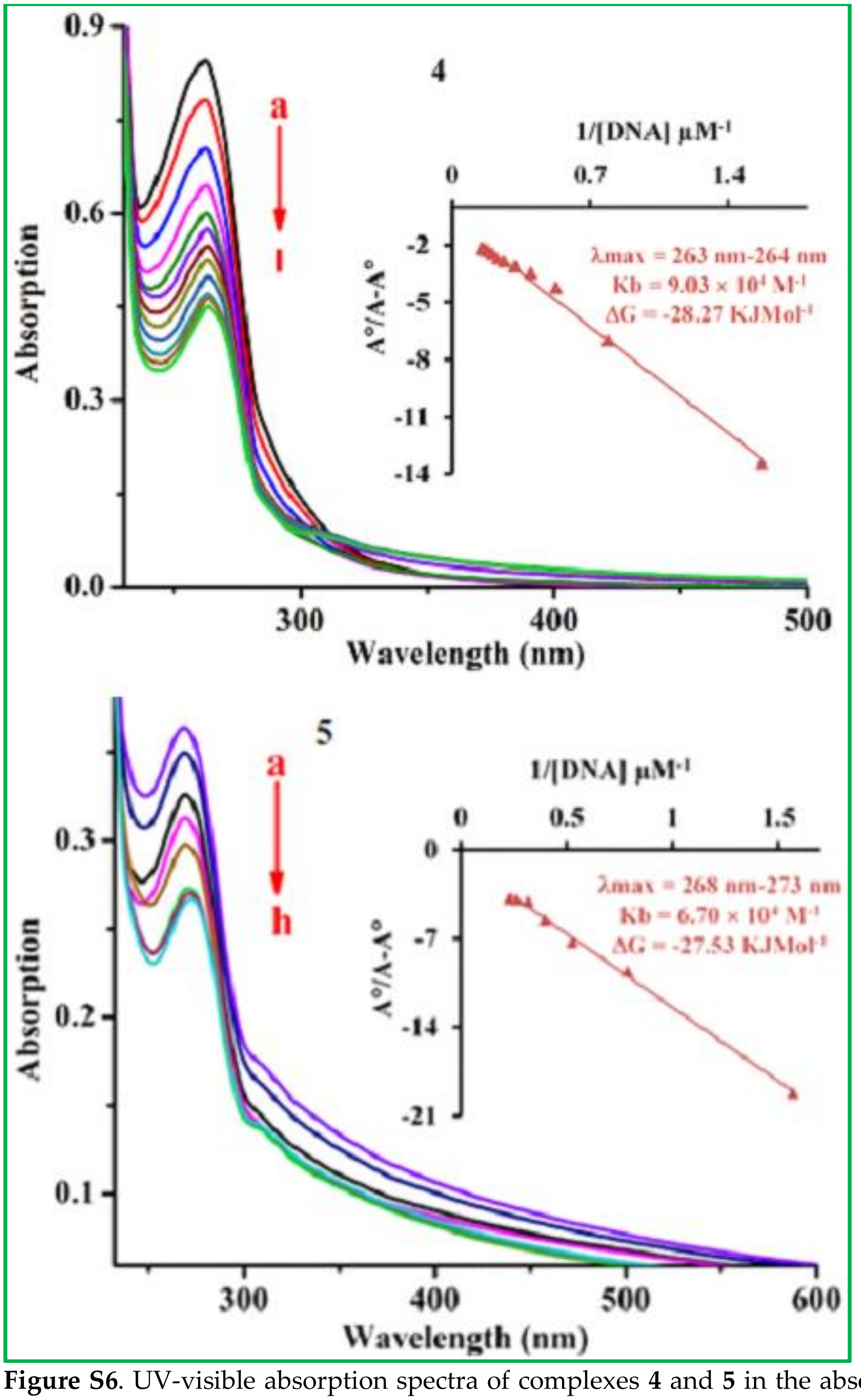

Figure S6. UV-visible absorption spectra of complexes **4** and **5** in the absence (a in each case) and in the presence of incremental addition, i.e., 0.63-5.04 μM (**4**) and 0.63-4.41 μM (**5**), respectively. Whereas the inside graph revels the values of Kb and ΔG calculated from graph plotted between the absorption and DNA concentration.

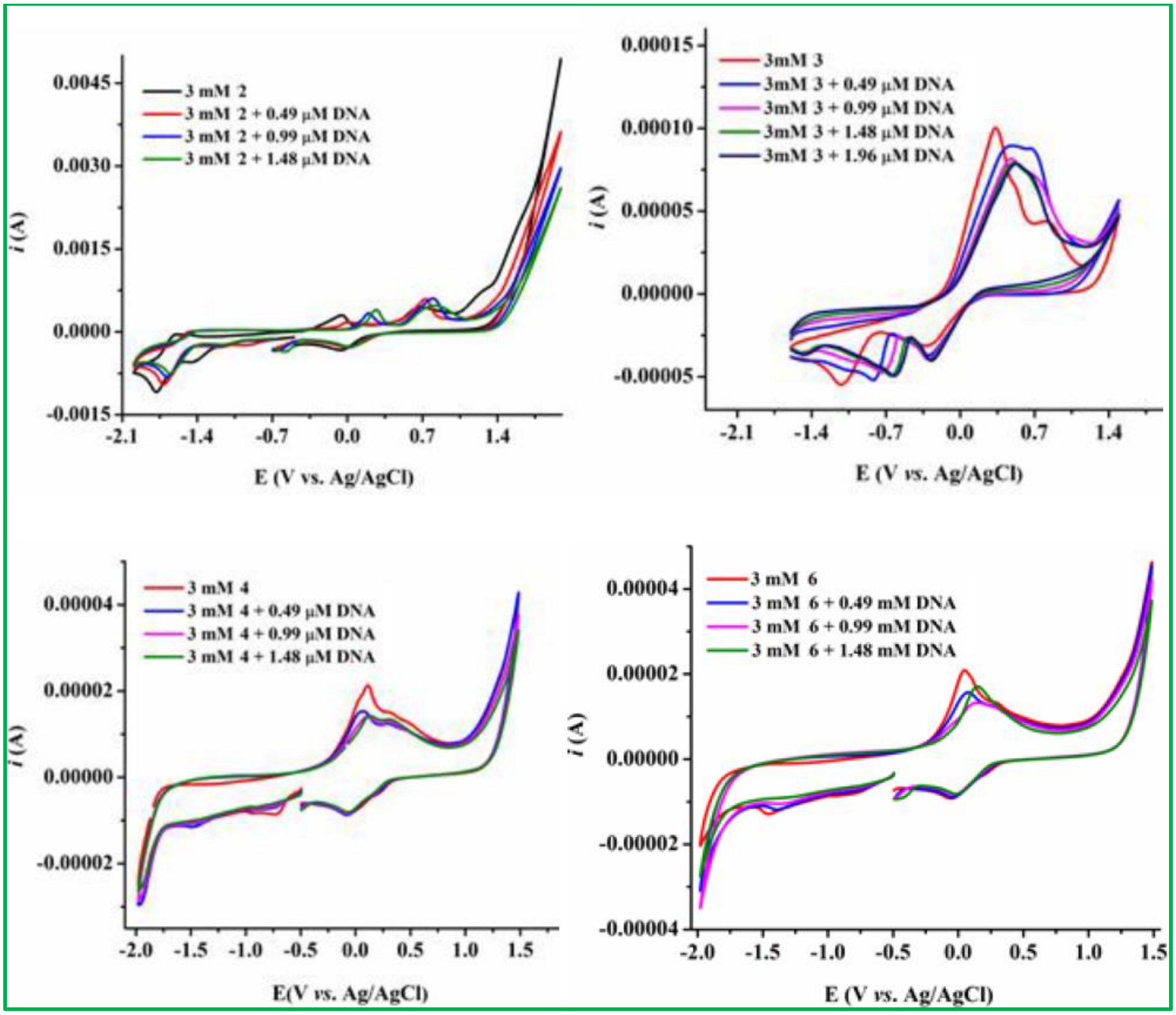

Figure S7: Cyclic voltammograms of the complexes **2**, **3**, **C4** and **6** in the presence of DNA

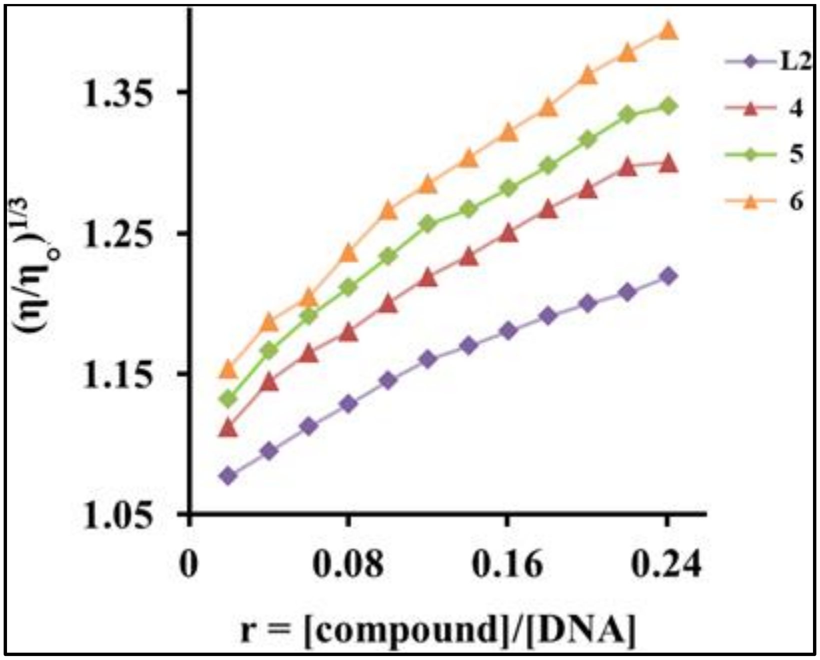

Figure S8: Graph presenting the effect on viscosity of SS-DNA by the incremental addition of L^2 and its complexes **4-6**
